# Supplementary material for: Discrete Mechanistic Target of Rapamycin Signaling Pathways, Stem Cells, and Therapeutic Targets
Source: Cells. 2024 Feb 27;13(5):409. doi: 10.3390/cells13050409 (PMC10930964; doi:10.3390/cells13050409)
Supplement: Supplementary file 1 [file cells-13-00409-s001.zip › cells-2850785-supplementary.pdf]

Supplement Table S1: Clinical Trials of PI3K/Akt/mTOR Signaling Inhibitors in Cancers

Inhibitors	Target	Cancer Type	Patients (n)	Clinical Trial Number(s)
Temsirolimus (Torisel)	mTOR	(I) Advanced malignancy<br>(II) Advanced cancer<br>(III) Head and neck cancer	(I) 47<br>(II) 44<br>(III) 42	**(I) NCT01050985 (completed, Phase I)<br>(II) NCT00877773 (early termination due to slow accrual, Phase I/II)<br>(III) NCT01172769 (completed, Phase II)**
Rapamycin (Sirolimus)	mTOR	(I) Advanced solid tumor and non-small cell lung cancer<br>(II) Advanced malignancy<br>(III) Head and neck squamous cell carcinoma	(I) 79<br>(II) 143<br>(III) 37	**(I) NCT05840510 (recruiting, Phase I/II)<br>(II) NCT01266057 (completed, Phase I)<br>(III) NCT01195922 (completed, Phase I/II)**
Everolimus	mTOR	(I) Well/moderately differentiated metastatic pancreatic neuroendo-crine tumors<br>(II) Advanced solid malignancies enriched for renal cell carcinoma	(I) 54<br>(II) 44	**(I) NCT02305810 (completed, Phase II)<br>(II) NCT01218555 (completed, Phase I)**
MLN0128	mTOR	(I) Advanced solid malignancy<br>(II) Metastatic castration-resistant prostate cancer	(I) 68<br>(II) 9	**(I) NCT01351350 (completed, Phase I)<br>(II) NCT02091531 (completed, Phase II)**
Ridaforolimus	mTOR	Advanced cancer	7	**NCT00836927 (early termination, Phase II)**
Dactolisib (BEZ235)	mTOR/PI3K dual inhibitor	(I) Advanced solid tumor<br>(II) Advanced pancreatic neuroendocrine tumor	(I) 33<br>(II) 31	**(I) NCT01343498 (completed, Phase I)<br>(II) NCT01658436 (completed, Phase II)**
Voxtalisib	mTOR/PI3K dual inhibitor	Advanced solid tumor	146	**NCT01390818 (completed, Phase I)**
Bimiralisib (PQR309)	mTOR/PI3K dual inhibitor	(I) Advanced solid tumor<br>(II) Recurrent or metastatic head and neck squamous cell carcinoma	(I) 70<br>(II) 8	**(I) NCT02483858 (completed, Phase I),<br>(II) NCT03740100 (early termination due to sponsor becoming insolvent, Phase II)**
Gedatolisib	mTOR/PI3K dual inhibitor	Triple negative breast cancer	110	**NCT01920061 (completed, Phase I)**
DS-7423	mTOR/PI3K dual inhibitor	Advanced solid tumor	42	**NCT01364844 (completed, Phase I)**
WX390	mTOR/PI3K dual inhibitor	Advanced solid tumor	38	**NCT06132932 (completed, Phase I/II)**
Buparlisib (BKM-120)	Pan-PI3K inhibitor	Advanced solid tumor malignancy	43	**NCT01470209 (completed, Phase I)**
AZD2014	ATP-competitive inhibitor of mTOR	High risk prostate cancer	23	**NCT02064608 (completed, Phase I)**
